# Formal support services and (dis)empowerment of domestic violence victims: perspectives from women survivors in Ghana

**DOI:** 10.1186/s12905-023-02678-5

**Published:** 2023-10-17

**Authors:** Ruth Minikuubu Kaburi, Basil Benduri Kaburi

**Affiliations:** 1 Department of Sociology, University of Ghana, Legon, Accra Ghana; 2 Ghana Field Epidemiology and Laboratory Training Programme, School of Public Health, University of Ghana, Accra, Ghana

**Keywords:** Domestic violence, Women survivors, Formal support services, Under funding, (Dis)empowerment, Revictimization, Ghana

## Abstract

**Background:**

As part of efforts to prevent violence against women, several countries have institutionalized formal support services including legislations to prevent, protect victims, and deter perpetrators of domestic violence (DV). Prior research on formal support service utilization shows that DV survivors do not get the necessary services they deserve. However, much remains to be known about the experiences of women survivors of DV who accessed a range of formal support services and how their experiences (dis)empowered them. Here, we assessed the experiences of Ghanaian women survivors of DV with formal support services vis-à-vis the provisions of the Ghana DV Act and insights of subject experts.

**Methods:**

From May to August 2018, we recruited a total of 28 participants: 21 women survivors of DV in Weija-Gbawe Municipality of Ghana, and 7 experts from the police, human rights, and health professions. We used two sets of in-depth interview guides: one to collect data on survivors’ experiences, and the second for the insights of experts. We performed summary descriptive statistics on survivors’ sociodemographic characteristics and used thematic analysis to assess their experiences of DV; and access to, patronage, and response of formal support services.

**Results:**

Of 21 DV survivors, 19 (90.1%) were aware of the existence of the DV law, however none was well informed of their entitlements. DV survivors have low formal education and are not economically empowered. Some DV survivors are revictimized in the process of accessing formal services. DV survivors expect the government to provide them with shelter, upkeep, medical, and legal aid. All the 21 survivors had at least one contact with a women’s rights organization and were knowledgeable of their supporting services namely legal services, temporary shelter, and psychosocial support.

**Conclusions:**

The experiences of DV survivors do not reflect the legal provisions of Ghana’s DV Act. Government under funding of formal services and negative gender norms are disempowering to survivors. NGOs are popular among women survivors of DV in Ghana for the education, legal, and material support they provide. A close collaboration between the government and NGOs could better mitigate DV in Ghana.

## Background

To curb the menace of domestic violence (DV), the international community over the years has made several efforts through world women conferences, human rights instruments, and United Nations declarations to eradicate violence against women. These concerted international campaigns have led to the creation of international gender rights norms such as the due diligence standard that prescribe how countries could protect their citizens against systemic gender-based violence and human rights abuses [1]. Several African countries including Ghana, have ratified international human rights treaties on the prevention of DV and violence against women more broadly [2]. Some of these treaties incorporate the due diligence standard that obliges ratifying countries to proactively prevent, investigate, and punish perpetrators of acts of violence against women in accordance with national laws [3, 4].

Prior to the emergence of international norms prohibiting violence against women, several African countries considered DV a private matter, thus not an issue for governments’ intervention [5–7]. However, actions aimed at revealing and fighting violence against women by Africa’s burgeoning women’s movement increased in the 1980s [8, 9]. This pressured governments to see DV against women as an issue of public concern. By the close of the 20th century, campaigns to eliminate violence against women became widespread with global attention drawn to the phenomenon of DV in the region [10–12].

Studies on the causes of DV in most African contexts offer that a mixed of individual, interpersonal, community and structural level factors often predict incidence of violence [13]. At the individual level, several factors such as age especially being young, alcohol abuse, depression and personality disorders, low income, educational levels, and having witnessed or experienced violence as a child often account for DV [14, 15]. On the other hand, marital disputes, weak family ties, enactment of traditional gender roles by couples, and economic constraints have been found to be contributors to DV within interpersonal relationships [16–18]. At the community and societal level, unaccountability of abusive behaviours, low social capital, traditional gender norms, and cultural norms supporting husbands’ use of violence to correct wives are factors that engender DV [13, 15, 17, 19, 20].

In 2007, Ghana passed the Domestic Violence Act (DV Act 732), hereafter DV Act, to create the legal basis to fight DV [5]. This DV Act specifically makes provision for filling complaints with the police and arresting perpetrators. It also makes provisions for criminal charges as well as civil procedures such as protection orders and the process for accessing these services. The DV Act also calls for the establishment of the Victims of Domestic Violence Support Fund to provide basic material and social supports systems in bringing relief to victims. Further, the DV Act explicitly gives a broad definition of DV, which embraces physical, sexual, economic, and emotional abuse. Compared to DV legislations of other African countries such as Botswana, Mauritius, Rwanda, and South Africa provide only police service interventions, Ghana’s DV Act has been described as progressive because it makes provisions for social and material support services in addition to services of the police and justice system [2, 5]. The DV Act notwithstanding, sociocultural, institutional, and economic factors that challenge availability and access to these services are rife at the national and local levels [21, 22].

Studies on government-sponsored protective services or formal support services including legislation against DV have reported that many women victims mostly turn to their informal networks first, wait long to report abuse to formal institutions, do not get the necessary services they deserve from these institutions, and may be blamed for the violence against them in the process of accessing formal support services [7, 21, 23–29]. However, to date, much remains to be known about the experiences of women survivors of DV in Ghana who sought formal support services and how their contact with these services (dis)empowered them in the process of seeking justice. Our study assessed and described the experiences of Ghanaian women survivors of domestic violence who contacted formal support services vis-à-vis the provisions of the Ghana DV Act and insights of subject experts.

## Methods

### Study design

From May to August 2018, we conducted a descriptive cross-sectional survey among women survivors of DV. We used in-depth interview guides to collect data on their perceptions, experiences, and perspectives on formal support services, and the availability and patronage of designated services. We also conducted key informant interviews among subject experts of government and non-governmental institutions concerned with protecting women against violence. We reviewed archival sources of secondary data from publicly available documents pertaining to violence against women to inform the interview guides and situate the data collected in context.

### Study setting

We conducted the study in Weija – an urban, and ethnically diverse community located about 27 km west of Accra – the national capital. According to the 2010 population and housing census of Ghana, Weija has a projected population of 15,892 – about 51.6% of which is female [30]. The town has a divisional police command with a Domestic Violence and Victims Support Unit (DOVVSU), and a municipal hospital that provides primary healthcare services. The town is commercially brisk with many shopping centers. It also has primary, secondary, and tertiary educational institutions.

### Sampling and recruitment of study participants

We recruited a total of 28 participants: 21 women survivors of DV in Weija and 7 experts with professions in DV, human rights, and health. There were three eligibility criteria for participating in the study. These were: women who had ever suffered DV, resident in the community 12 months or longer prior to the start of our study, and a history of seeking formal DV support services on at least one occasion. To get access to our study sample, we contacted leaders of the three local non-governmental organizations who work with abused women and provide counselling service to survivors of DV in the community. We used convenient sampling to recruit women survivors who showed up, met the selection criteria, and gave consent to participate in the study. We stopped recruiting participants on reaching theoretical saturation.

Three of the seven respondents to the key informant interviews represented three departments namely, the Department of Social Welfare, the Domestic Violence and Victims Support Unit (DOVSSU), and the Commission on Human Rights and Administrative Justice (CHRAJ). The remaining four experts were a medical officer, and three women’s rights advocates. The women’s rights advocates represented three non-governmental women’s rights organizations namely, Ark Foundation, International Federation of Women Lawyers (FIDA-Ghana), and Women Alliance, Ghana. We purposively selected these key informants based on their professional expertise and experience in DV support services in Ghana.

### Data collection

We used semi-structured interview guides to collect data. We adopted two separate interview guides from the Ghana Domestic Violence Report [31]: one for the women survivors of DV and the other for the experts. The interview guide for women survivors of DV contained variables on sociodemographic characteristics, their knowledge of the DV Act (732) 2007, awareness of the scope of formal support services, their perspectives on these support services, and how survivors exercised their rights of seeking support and redress from government agencies. We conducted interviews with the women survivors in *Twi* - the most widely spoken language in the community. We first wrote the interview guide in English language and translated it to *Twi* with assistance of two native speakers. We used back-translation reiteratively during transcription and analysis process to ensure that the accurate meaning of the original responses of participants were maintained. The durations of interviews with DV survivors averaged 35 min. We conducted the expert interviews in the English language; each lasting an average of 45 min. The in-depth interview guide for the experts included questions relating to challenges of service provision, awareness of domestic violence act, awareness of various support services available to survivors, and survivors’ attitude towards formal support services. We reviewed records of relevant secondary data and described the disconnect between government advocated directives on DV and the experiences of DV survivors.

### Data analysis

We described survivors’ sociodemographic characteristics using summary descriptive statistics. We performed a descriptive thematic analysis on the qualitative data from participants’ responses based on predetermined themes namely, perceptions, experiences, the government’s responsibilities to DV survivors, the availability of expected services, and their experiences with access to formal services.

### Ethical considerations

The Ethics Committee for Humanities at the University of Ghana granted ethical approval for the study. We explained the background and purpose of the study to the participants. They were made aware that no form of compensation would be given for participation, but that their participation could possibly benefit the fight against DV. We also made the participants to understand that participation in the study was voluntary and that they could withdraw at any point or refuse to respond to any questions without the need to explain themselves or fear of any repercussion. All respondents signed a written informed consent for participation.

We protected the privacy and safety of the respondents by using an inner room at the offices of the Department of Social Welfare at the Weija-Gbawe Municipal Administration. We also made provision for a counsellor to be at hand to support any participant who may experience emotional breakdown. We stored the hard copies of completed interview guides under lock and key, and the recordings and transcripts of the data under password. We pseudonymized the data by keeping personal identifiers separate from the responses. Only the authors analysed the data.

## Results

### Sociodemographic characteristics of women survivors of domestic violence

A total of 21 women survivors of domestic violence participated in our study. Their ages ranged from 23 to 54 years; with a median age of 42 years. Of these 21, 18 (85.7%) were married, 13 (61.9%) had 3 to 5 children (Table 1). The highest level of formal education among them was senior high school; with 3 (14.3%) attaining this. Majority – 14 (66.7%) engaged in petty retail trading. About half of them 11 (52.4%) depended entirely on their abusers for upkeep (Table 1).

**Table 1:** Socio-demographic characteristics of women survivors of domestic violence, Weija-Gbawe Municipality, Ghana

Characteristics	Number (n = 21)	Proportion (%)
**Age**		
20–29	3	14.3
30–39	5	23.8
40–49	9	42.9
50–59	4	19.1
**Marital Status**		
Married	18	85.7
Single	3	14.3
**Number of Children**		
0	1	4.8
1	4	19.1
2	3	14.3
3	4	19.1
4	4	19.1
5	5	23.8
**Educational Level**		
None	6	28.6
Primary	2	9.5
Junior high school	4	19.1
Middle school form 4	6	28.6
Senior high school	3	14.3
**Occupation**		
Petty trading	14	66.7
Food Vending	4	19.0
Hair Dressing	1	4.8
Unemployed	2	9.5
**Breadwinner**		
Partner	11	52.4
Survivor	6	28.5
Both partner & survivor	4	19.1
**Organizational Membership**		
Church Women’s Fellowship	9	42.9
Church Choir	3	14.3
None	9	42.9

### Perspectives of women survivors of DV on formal support services

Survivors’ perceptions of legislation and formal support services fell into four categories viz. survivors knowledge of the scope of services and utilization; survivors experiences of accessing formal support services; challenges associated with utilization of formal support service; and (dis)empowerment and agency of survivors accessing formal support services.

#### Survivors’ knowledge of the scope of support services and utilization

There were three main categories of formal support services that were oversteered by government agencies. These were the criminal justice services, social services and basic material support, counselling, and mediation services from quasi-judicial institutions. These services were complemented by a range of support services rendered by civil society organizations approved by the government.

#### The criminal justice services

Review of relevant secondary data showed that the criminal justice services comprised arrest and detention of abusers by the police and the adjudications of DV cases by the law courts and family tribunals. DOVVSU of the Ghana Police Service was reconstituted as a gender neutral entity to tackle gender-based violence from the then Women and Juvenile support Unit [WAJU] in 2005 [5]. This unit is the main point of call for survivors when a case of abuse is reported to authorities. It also served as a point of referral to and from other services within the formal support system [32]. Specifically, DOVSSU received and responded to complaints, conducted investigation into cases, provided temporary shelter, referred victims for medical, legal, and counselling services. It also referred dockets to the Justice Department for advice on prosecution [33, 34]. The DV law in Ghana also made provisions for civil protection orders to protect survivors of violence.

The courts handled DV and other violence against women specified by law. In order to swiftly respond to DV, the judiciary service operated human rights, gender-based and sexual offences courts to speed up the adjudication of cases. The justice system in the country also made provision for the family and juvenile courts at the district court level to handle mild cases of DV and other non-violent family disputes bordering on economic neglect and child custody. These courts used alternative dispute resolution (ADR) methods to settle cases. The family courts also had jurisdiction to deal with criminal cases and civil protection orders under the DV Act. Of the 21 survivors, 13 (61.9%) were aware of the role of the police and courts in the provision of formal support for them.

Four (19.1%) of the survivors filed complaints at DOVVSU that led to arrests of their abusers (Table 2). They complained that they experienced difficulties in their attempts to access justice at the law courts for lack of money to file their cases, pay for medical reports, and fund legal representation. Of the 21 DV survivors, 19 (90.1%) were aware of the existence of the DV Act, but none of them knew about the protection and occupation orders it provided as an avenue of seeking protection.

#### Social services and basic material support

Support services under this category included provision of reception shelters, medical care, skill training, rehabilitation, and reintegration with families. These services were to be provided free of charge under the domestic violence fund. The establishment of the DV fund under the law was equally challenged by low government budgetary allocation to the sector. Of the 21 survivors 5 (23.8%) were aware of the scope of social and material support services, and14 (66.7%) of them sought medical care from health facilities. Of the 14 who sought medical care, 1 (7.1%), had valid health insurance. The remaining had to make out of pocket payments - some with the help of friends and relatives. A medical report costed a minimum of 100 Ghana cedis (20 USD in 2018), provided medical evidence of the abuse in court, these survivors perceived the fee expensive. On different occasions, police officers accompanied three of the survivors to the hospital. On one such occasion, a police officer had to pay the medical bill of one of the survivors. The law requires the police to assist victims to obtain medical care, but is not explicit on the specifics of the assistance such as accompanying victims to hospitals and paying their bills.

All the DV survivors and subject experts agreed that paying for medical care and medical reports before a case can be booked in the law courts is a disincentive for the pursuance of DV cases. The medical report is a mandatory requirement particularly for physical assault including sexual abuse without which the police cannot prosecute the case. There was no form of specialized or priority services for survivors of DV who reported at the health facilities.

The Department of Social Welfare (DSW) is a government agency mandated to provide psychosocial counselling and shelters for abused women. Of the 21 survivors, 5 (23.8%) had knowledge of the range of services provided by this agency and 10 (47%) utilized psychosocial counselling services. Nevertheless, the provision of shelters remains non-existence due to inadequate funding to operate them. Some survivors indicated that the threat of homelessness delayed their decision to seek help from formal support services. A survivor who got separated from her husband after reporting him to the police and is now lodging with her sister shared her frustration:

*If my sister did not take me in, I don’t know what would have happened to me. However if government provides a place where people in my situation can lodge for the meantime whilst they reorganize their lives, it will help. (Survivor 1, Interview, May 3, 2018)*

In the absence of shelters for battered women, the DSW provided psychosocial counselling, conduct physical and medical needs assessment and refer survivors to other support services including the services of partner civil society or non-governmental organizations. The DWS also provided counseling and mediation services particularly within the family tribunals. Specifically, the DSW mediated DV incidents that were considered civil cases, as against criminal. These case bordered on economic abuse including child maintenance and paternity disputes among couples.

Mediation and counseling services from quasi-judiciary agencies.

The Commission on Human Right and Administrative Justice (CHRAJ) also provided mediation and counseling services. Of the 21 survivors, 1 (4.7%) reported knowledge of the role of CHRAJ in supporting abused persons, and none utilized any services from this agency. CHRAJ is a quasi-judicial institution created under the 1992 Constitution to help promote transparency and public accountability in the area of human rights protection.

The DV Act (Sect. 24) made provisions for the court in a criminal trial of DV cases to promote reconciliation by referring some cases to mediation under certain circumstances. These circumstances included when the violence is not aggravated or does not require a prison sentence more than two years, the victim offers to have the case settled out of court, and when the court had the opinion that the case can be resolved peacefully through alternative disputes resolution (ADR). Following a complaint of human rights abuses, officials of CHRAJ invited both the complainant and respondent to talk out their issues. As a neutral facilitator, the officials helped the parties to negotiate a mutually beneficial agreement for an amicable settlement. Though these settlements or agreements are not legally binding and a party can violate it without any consequences, CHRAJ was still a point of call for some DV victims. When parties do not reach an agreement, they are referred to the judicial system for redress.

#### Government approved support services provided by civil society organizations

Our study found that civil society organizations including non-governmental women’s rights organizations also played an important role in the provision of services to DV survivors. The services provided by these entities were supplementary to the formal support services. The range of services rendered included training and advocacy on DV prevention, psychosocial and legal counselling, education on gender-based violence, skills training, and provision of temporal shelter for survivors of DV.

The International Federation for Women Lawyers (FIDA-Ghana) provided free legal aid to women survivors, initiated research into socio-legal issues affecting the status of women and children, trained and sensitized stakeholders and the public on gender-based violence, served as referral centers for DV survivors from the police, CHRAJ, and DWS for legal assistance. Of the 21 survivors, 2 (9.5%) sought assistance for redress from FIDA. All the 21 survivors indicated that at one point, they had contact with these women’s rights organizations and were knowledgeable of the services they provided. At the time of this study, FIDA-Ghana also operated a flagship legal literacy and capacity building program that trained community members to serve as paralegals to DV survivors and other vulnerable groups at the community level. The project also offered legal training on handling gender-based violence with police officers in some selected police stations.

#### Survivors experiences of accessing formal support services

Severity of abuse, social, and economic obstacles informed preference for specific services.

Of the 21 survivors – 16 (76.2%) experienced physical violence (Table 2). Four of these 16 survivors (25%) of physical violence experienced sexual violence in addition. Severe physical violence was a major trigger to seek formal support services. Other forms of violence included verbal/psychological abuse, and economic abuse in the form of financial neglect. Nearly all 20 (95.2%) were abused by their intimate partners. The remaining survivor was abused by her in-laws. Counselling was offered to 10 (47.6%) of the survivors (Table 2).

**Table 2:** Experiences of women survivors with formal support services, Weija-Gbawe Municipality, Ghana

Types of experience	Number (n = 21)	Proportion (100%)
**Form of domestic violence experienced**		
Physical violence only	12	57.1
Physical and sexual violence	4	19.1
Verbal abuse	1	4.8
Financial neglect	4	19.1
**Knowledge of existence of DV Act**		
Yes	19	90.5
No	2	9.5
**Knowledge of provision of protection orders**		
Yes	0	0
No	22	100
**Knowledge of forms of support services**		
Criminal justice system	13	61
Free social and material support (DSW)	5	23.8
CHRAJ	1	4.8
FIDA (government approved services)	2	9.5
**Formal support received**		
Arrest, detention and cautioning	4	19.1
Counselling	10	47.6
Summoned and cautioned	1	4.8
Failed Arrest	2	9.5
None	4	19.1
**Survivors’ satisfaction with Support**		
Satisfied	6	28.6
Not satisfied	14	66.7
On going	1	4.8
**Outcome of support**		
Violence did not stop	13	61.9
Violence stopped	7	33.3
Support is on going	1	4.8

Survivors who received counselling service indicated that they felt better afterwards even though counselling alone did not stop the abuse. Survivor 3 who reported her ex-partner to the department of social welfare for economic abuse and the refusal to pay child maintenance shared her experience:

*At the social welfare department, I was advised to avoid situations that will lead to fights resulting in my child’s father not giving money. The officer also told me to engage in some economic activity to reduce financial pressure on him (ex-partner). My ex was also advised to take up his responsibility until our child becomes 18 years. He agreed but did not change his behaviour thereafter. (Survivor 3, Interview, May 3, 2018)*

Four of the 21 survivors (19%) confirmed that the perpetrators of violence against them were arrested and detained by the DOVVSU of the Ghana Police Service. In cases of severe physical and emotional abuses, survivors in our study obtained support from their friends or relatives and advice on decision to report the abuse to the police. Survivor 4 who had endured physical and sexual violence from her husband for many years had the support from family members when she decided to report him. Her decision to report her abuser led to a separation, and the case was pending adjudication at the time of our study. She recounted that:

*Reporting the case has led to our separation. I am living with a friend for the meantime. I thank God that my children are now adults. (Survivor 4, Interview, May 3, 2018)*

Aside fear of loss of life, severe and prolong violence was also a driver of the decision to report the abuse to authorities. In such cases, the informal networks of survivors (e.g., friends, family members, and pastors) supported them to report abuses to the police. The support from these agencies ended the violence for seven (33.3%) of our study participants (Table 2). Survivor 8 who had relief from her abuser after reporting him to the police narrated that:

*when the police arrested and asked him to report to the police station every two days, he stopped abusing me. So far, I am satisfied because he has stopped beating me. (Survivor 8, Interview, May 3, 2018)*

### Challenges associated with utilization of formal support services

#### Delays and need for frequent follow-ups

Survivors in our study mentioned delays especially with in the criminal justice system as the reason why they dropped their cases. Survivor 6 suspected that her abuser might have induced the police to dismiss the case following his arrest and cautioning. According to her, on his release, he abandoned her and the family. She shared her experience:

*The police made me go to the hospital and they arrested him. After he was released later, he packed his clothes and left without telling me anything. Subsequent follow up with the case officer was futile, as his whereabouts was not known. Maybe he paid money to have the case dismissed. (Survivor 6, Interview, May 3, 2018)*

Delay in getting services was reported as a common cause of dissatisfaction with formal support services. Whereas six (28.65%) were satisfied with the support offered by service providers, 14(66.7%) were not satisfied with the services. Narrating her dissatisfaction with the formal support services, Survivor 7 recounted as follows:

*When I reported the case at DOVVSU, there were delays and this necessitated frequent follow-ups. I did not have money to continue the case to the court, so I stopped pursing the issue. My marriage is unstable now, the abuse has not stopped, and he is angry that I reported him to the police. (Survivor 7, Interview, 3 May 3, 2018)*

A women’s right advocate lamented on the delays in the adjudication of DV cases in the courts:

*The slow pace of obtaining justice on DV cases is frustrating. When you report a case, it takes a long time to be adjudicated…if you don’t follow up repeatedly, the case will come to naught. (Women’s rights advocate 1, Interview, June 12, 2018)*

#### Survivors’ lack of economic empowerment

Most DV survivors in the study were not economically empowered. This countered their efforts at seeking medical care, and medical reports. Section 8 of the Act makes provision for free medical care for DV survivors, but this is not provided for and hence, not complied with at the point of services. In adequate funds for this sector has not created an enabling environment for implementation of this provision. Consequently, survivors express the view that the free treatment and medical reports need to be urgently implemented or at worse medical bills to be charged on abusers. Survivor 9 also made this proposal to government as a way of deterring abusers in the following statement:

*The government should educate the men and let them know that there is a law protecting women from domestic violence. If they do not comply with it…they would be arrested, pay medical bills, and punished severely. (Survivor 9, Interview, May 3, 2018)*

DV survivors perceived hospitals as one places where they have temporary shelter devoid of the risk of encountering the perpetrators. They were of the conviction that, attending public hospitals in their time of distress is important and should not attract fees, whether for treatment or medical reports.

#### Survivors lack of comprehensive knowledge on the scope of support services

Survivors were not able to seek all the range of services available to them under the DV Act because of their limited awareness of these services. Hence, in expressing their expectations, some survivors called for the police in cases of arrests and subsequently release to secure a bond of non-violent behaviours (protection order) from their abusers, even though this service is stipulated in the Act. Those who suffered financial neglect expected a bond on their partners to assume their financial responsibilities to the family.

#### Institutional constraints

The efforts of survivors to access justice were thwarted by institutional constraints. This often result in delays attributed to several factor such as lack of central government budgetary allocation for service provision, lack of logistics by officials to carry out their mandate and inadequate trained official specialized in handling gender-based violence. A women’s Right advocate at FIDA-Ghana stressing on these constraints have this to say:

*They (police) will help but the speed with which they do that is not fast. We partner with some police stations and offer training on the handling of DV cases. But we do this on piecemeal basis …I wish this could be done on a larger scale, but it is not feasible because of limited funds at the disposal of NGOs. Ideally, government should offer these training in larger scale to help the police to be more responsive in handling these cases. (Women’s right advocate 2, Interview, 14 June 2018)*

This state of affairs negatively impacted on survivors desire to see the police arrest and prosecute abusers (for severe cases of physical and sexual abuse), fine, and caution where necessary. However, prosecution in severe cases was hindered among survivors who sort for this service. This was partly due to ineffective law enforcement in some cases, the unprofessional conduct of officials in the forms of trivializing domestic violence cases, accepting inducements from abusers and acting cold and unsupportive of survivors’ case. Similarly, an officer from the DSW stated that inadequate funding hinders the provision and maintenance of government-run shelters (Officer, Department of Social Welfare, Interview, June19, 2018). Our records review also found that the government budgetary allocation for the Ministry of Gender, Children and Social Protection for its expanded role was under 1% of the total national budget as of 2015 [35].

One of the three women’s rights advocates indicated that police trivialization of DV and the cold and sometimes harsh reception of victims at the police stations instantly discouraged women reporting DV. Oftentimes, this discouraged survivors from pursuing the case even if they succeeded registering it. She shared some experiences of survivors who reported at the police station to file a complaint and some of the responses they got were:

*“What did you do before he beat you… go home if he beats you again then you come” and*.

*“You are reporting your husband to the police? He will leave (divorce) you”. (Women’s rights advocate 3, Interview, June 20, 2018)*

### Survivor (dis)empowerment and agency in seeking formal support services

Survivors in our study find that positive attitude from officials and support from their informal networks to their decision to seek formal support services empowering. Survivors also appeared to have internalized local and national campaigns against violence with emphasis on abuser accountability. This is reflected in the majority 19 (90.5%) of our participants indicating knowledge of the existence of the DV to punish abusers if victims reported the abuse, even though they were not conversant with the specificities of the provisions of the law itself.

An officer of the department of social welfare weighed in on DV survivor’s awareness of formal support services stating that:

*Previously, fewer DV survivors reported abuse, but now more of them are reporting. This is because of education on radio, TV, and community outreaches by women’s right organizations. Even with this education, family and friends discourage some women from reporting abuse to us. It is only on few extreme cases of violence that survivors’ informal network supports their decision to report the perpetrator. (Social welfare officer, Interview, June 19, 2018)*

This awareness notwithstanding, all survivors indicated that their initial point of call to end the abuse was to their informal social network. Also, they all indicated that their first decision to access formal support services was informed by their contact with media and community outreaches of DV activists and backed by some form of support from at least one family member or friend.

Though some survivors in the study still faced resistance from their informal networks in reporting abuses, majority of them 12 (57.1%) reported some instances that they were \ supported financially and psychologically by relatives and even law enforcers to report their abuse. In all these instances, the abuse was severe and habitual. For instance, a survivor’s sister encouraged her to report the abuse, and later supported her accommodation needs. Another Survivor also reported that she was motivated to continue the filing of a complaint against her partner after she received encouragement and support from a police officer. She narrated that:

*When we got to the hospital, I did not have enough money to pay for the services. Had it not been the police officer who paid the bills, I would not have been attended to and as such couldn’t have filed the case. (Survivor 2, Interview, May 3, 2018)*

A significant finding is that psychosocial and financial supports from victims’ informal networks as well as law enforcers were drivers for decision to report the abuse to the criminal justice system. Nevertheless, some survivors of the study still reported revictimization and pressures from their families to some extent. Of the 21 survivors 6 (28.5%) felt disempowered by discouragements from their informal networks and negative attitude of officials. These survivors reported exercising their agency by defying these setbacks and reported their abuse. Whilst others 3 (14.2%) issued threats to the abuser. Threats of reporting alone did not stop the abuse even though they sent a signal to the abusers that survivors were aware of the avenues for redress. Defiance and issuance of threats were ways that some of the survivors negotiated setbacks that were disempowering to them in seeking state support services.

### Insights and perspectives from experts

The expert interviews revealed that the implementation of the DV Act had been problematic. This had to do with a disconnection between the DV law and its actual implementation. For instance, Sect. 29 of the DV Act makes provision for the establishment of victims of domestic violence support fund intended to provide basic material needs and support for victims. This fund was not operational. Some of the identified impediments that created this disconnect included lack of awareness of the Act and its provisions, inadequate funds for the implementing agencies, and inadequate personnel with appropriate specialized skills to handle domestic violence cases. A women’s right activist who bemoaned the lack of knowledge of the protection and occupation order had this to say:

*Even professionals including police officers and social workers are not conversant with the protection and occupation order and procedure for their application as protection strategies for survivors of DV. (Women’s rights advocate 2, Interview, June 14, 2018)*

A police officer at DOVVSU stated that, inadequate logistics hinders the effectiveness of their services to the public. In explaining the challenges of DOVVSU further, the officer stated that:

*Lack of financial resources to run the office negatively affect service delivery to the public. Other challenges we face include inadequate training of personnel on effective prosecution of domestic violence and sexual and gender-based violence cases, and also heavy workload. (Police officer, Interview, May 30, 2018)*

A women’s right advocate pointed out that getting a medical report at the right time is challenging to most women; and delay in getting the report because of financial difficulty tempers evidence (women’s rights advocate 2, Interview, June14, 2018).

The medical report fee commits a medical officer to the case and is meant to defray costs of transportation should the officer have to appear in court in person to give testimony during hearing of the case. A medical officer interviewed on the rationale of the fee charged explained that:

The issue is that, if the government takes up the costs incurred in terms of transportation and other related costs for medical officers to appear in court to testify, then the medical fee would be waived for DV survivors. (Medical officer, Interview, June 4, 2018).

All the women survivors in the study and experts agreed that paying for medical care and medical reports before a case can be booked is a disincentive for the pursuance of DV cases.

The interview with CHRAJ official explained that mediation and counselling by psychologists are often used to resolve milder cases of abuse, with an aim of promoting reconciliation. However, in cases of extreme physical violence the law was applied (CHRAJ official, Interview, June, 12 2018). The process and conditions for the referral of mild cases is well prescribed at Sect. 24 of the DV Act.

In the case of providing protective services to complement formal support services, The Ark Foundation – a Christian based non-governmental organization had a shelter for battered women. However due to financial constraints, the organization could no longer run the shelter at the time of this study. Hence, the organization resorted to shelter provision with support from other partners who sponsor the upkeep for persons they refer there (Women’s rights advocate 1, Interview, June 12, 2018).

According to a women’s rights advocate, some external family members of the survivors mostly prefer counselling or verbal cautioning of the abuser compared to prosecution in less severe instances of abuse. The advocate explained that the family members prefer this option because:

*Arresting and prosecuting often lead to separation and divorce, which is detrimental to the family if they have children and especially if the abuser is the sole bread winner. (Women’s right advocate 3, Interview, June 20, 2018)*

These pressures often led to complainants withdrawing the case before adjudication even began. For example, Survivor 5 from our sample was pressurized by family members to drop a complaint filed at DOVVSU. She revealed that:

*Our family members advised me not to pursue the case because of our young children. I had to drop the case because I would have been tagged disrespectful to them if I did not. (Survivor 5, Interview, May 3, 2018)*

## Discussion

Our study sought to assess formal support services from the perspectives of women survivors of DV in Ghana who accessed them and how their experiences with these services (dis) empowered them in seeking justice in the Weija-Gbawe municipality. A majority of the DV survivors in our study were married, attained basic formal education, young, and not economically empowered - sociodemographic profiles consistent with survivors of DV survivors in Malawi [36], and earlier reports from Ghana [37, 38]. Women survivors of DV who are not economically empowered in Ghana face financial constraints in accessing formal support services. Advocacy for such women to be economically empowered either through the attainment of higher formal education or informal vocational skills could positively influence the outcome of their contact with formal support agencies to a large extent [39, 40].

Other studies also call for changes in societal norms to promote equality among men and women [41, 42]. For instance from vulnerability theory standpoint, Fineman [43] and Kohn [44] offer that there is the need for the governments to provide equal access to public institutions and laws that distribute social goods such as healthcare, employment, and security. This call is timely as it has been well documented in the Ghanaian society and similar contexts that gender gaps exist in access to education, income, access to wealth from inheritance, access to health services, and household decision-making [45–47].

Several studies of the DOVVSU as a specialized police unit find that, due to severe institutional constraints such as heavy workload, inadequate trained personnel and lack of logistics to operate, DOVSSU officials are not able to provide satisfactory services to DV victims [5, 6, 48]. This corroborates our finding that government institutions such as DOVVSU that have the mandate to provide support services are beset with logistical constraints and inadequately trained personnel and hence, are unable to function as stipulated by the DV Act. Generally, the literature on legislation against DV often shows a disconnect between the law and its implementation [2, 49]. Governments more often do not follow up with adequate funding, infrastructure, human resource, and logistics to guarantee successful enforcement after passing DV laws. As a constitutional democratic country, the 1992 constitution of Ghana mandates government officials to ensure that the fundamental human rights of all citizens are protected. Additionally, countries’ ratification of various human right treaties at the United Nations and the regional level makes it imperative for authorities to take active measures to protect the human rights of citizens. In this regard, Ghana has not been successful in protecting DV victims. In our study, the social and material support for medical care and shelters were not available even though the law makes provisions for such services. For example, other studies have shown that affordable and timely medical service not only provide verifiable evidence of abuse but also save lives in cases of severe physical injuries [50, 51]. Similarly, Meyer [52], and Stylianou and Pich [53] underscore the critical role of shelters for abused persons fleeing their abuser. The absence of shelters and medical cover undermines efforts to protect DV survivors who sought help from formal support services in this study. Given the crucial role that medical services and shelters play in filing a case against perpetrators, several studies reiterated the need for authorities to make these services free or affordable [32, 53, 54].

Some civil society and non-governmental organizations including faith-based institutions have stepped in to provide basic social support services for survivors. Even though these benevolence institutions also has faced financial difficulties, their contribution to the fight against domestic violence in Ghana is notable. This goes to underscore the need for governments to collaborate with non-governmental and civil societies organizations in the fight against gender-based violence. Through the pulling of resources together, the government and non-governmental actors could possibly enhance protective service delivery to DV survivors.

It is mandatory that once a DV victim files a complaint of physical and/or sexual abuse with the police, she is referred to a health facility to obtain a medical report detailing her health condition related to the violence. This report serves as proof of the abuse without which, the police may decline proceeding with the case. For example, Cantalupo et al. [55] report that prosecutors at the Attorney Generals’ Office cannot effectively handle cases without the medical testimony. A prosecutor in their study underscored the importance of the medical testimony to this effect: *“cases with the absence of medical testimony can result in less compelling, unarguable, or even damaging evidence…*[55] *.* Due to inability to afford medical reports, the survivors of DV in the study were discouraged from pursing criminal justice services. Depending on the peculiarity of the case, medical officers must necessarily testify at trials. This further increases the costs of obtaining the medical reports because, the expenses anticipated in respect of court appearances by the medical officer are factored in the total cost of the medical report [49]. These challenges could partly explain why none of the survivors at the time of participating in this study had redress for their cases at the law court.

Court-guaranteed protection or occupation orders to protect survivors are not utilized for reasons including ignorance of their existence by both law enforcers and survivors. A study by FIDA-Ghana on why few women survivors of DV apply for the protection and occupational orders in Madina (a comparable community in Accra) corroborate our findings [56]. Similarly, Darkwah and Prah [49] also found that some police officers were not conversant with the DV Act and its provisions. Aside from the lack of knowledge of the protection orders, reports on their effectiveness to prevent re-abuse are mixed. Carlson et al.’s work on women who were issued with protection orders found that those with very low socioeconomic status (SES) and African American women were more likely to find protection orders ineffective in preventing re-abuse [57]. They argued that out of economic necessities, women of low socioeconomic profiles could not afford to separate from their abusers on whom they depend for sustenance. This draws attention to the fact that even if our study participants were aware of and sought protection under protective orders their re-abuse will likely not end. In effect, even though these orders are civil and preferred by some survivors, they cannot adequately protect them due to sociocultural and economic factors.

As similar studies in Nigeria and South Africa show, severe forms of physical and emotional violence are predictors of help seeking behaviors among women victims of DV in our study [58, 59]. This finding shows that women victims reporting abuse to government officials are mostly at their wit’s end and extremely expectant of the needed support services.

African countries such as Ghana need to go beyond legislation, by instituting robust social welfare systems and actively scrutinize the role sociocultural practices play in conferring privileges and power between men and women, which to a large extent are deliberately or inadvertently backed or at least condoned by the law and the justice system.

Financial constraints are disempowering to survivors following up on their cases. Again, the withdrawal of cases due to extended family interference mostly on claims that the criminal justice regime is retributive also worked against the agency of some survivors. There is a growing literature that advocates for a policy shift by governments to popularize restorative justice avenues already existing within the family courts system [60, 61]. This could be useful to some survivors seeking redress for their abuses in low income setting and could address gaps in current gender-based violence policies and make them inclusive for vulnerable population. This notwithstanding, it can be argued that the mediation component of the DV act, though it gives room for family reconciliation, its flip side could be perpetuation of habitual non-violent to milder forms of violence. It can come across as not being deterrent enough for abusers. It could also expose survivors to more violent and could be a disincentive in accessing formal support service.

Our findings suggest that the emergence of the due diligence standard including DV legislations in patriarchal societies such as Ghana have resulted in empowerment of the abused persons. Despite the inadequacies of formal support services for survivors in the study, the culture of silence and secrecy that shrouded DV seems to be eroding gradually - making women increasingly able to assert their rights to protection beyond their informal networks. This positive shift in agency corroborates evidence from South Africa that contends that social policy shift in deconstructing DV within a private/public sphere could lead to rapid progress on DV prevention [62].

Unfortunately, our findings show that contacting the criminal justice system did not end the violence for majority of women survivors of DV. Some survivors suffered strained relationship with their partners, homelessness, and revictimization due to negative attitude of service providers and their informal networks following reports of abuse. These findings underscore ways that people reporting abuse end up being disempowered. Some researchers have argued that legislations alone do not prevent violence [63]. Such voices called for the need to couple legislation with a robust activism with people at the community level in addition to the provision of basic social and material support for vulnerable populations seeking relief from abuse [9, 63, 64].

All survivors who reported for formal support service received counselling. Generally, counseling sessions for our study sample were able to ease distress from depression, anxiety, posttraumatic stress symptoms, guilt, and shame that often accompany victimization as reported by Bennett et al., Mcleod et al., and Sullivan and Rumptz [see 65, 66, 67]. This service was widely available even in low income setting and helped maintain survivors’ sense of self-esteem and well-being. Counselling as a form of psychosocial support was predominant because of the relative availability of professionals among services providers in the country. It is a relatively cheaper form of support offered by the formal support services. Even though counseling alone was incomplete for addressing the needs of survivors, some psychotherapists note counseling as having the potential of empowering survivors of DV. Counseling helps survivors to externalize their problems, whilst at the same time harness their ability to internalize their personal agency to cope and to take measures to prevent their problems [68]. Defiance and issuance of threats are ways in which DV survivors in Ghana have negotiated setbacks that are disempowering to them in terms of accessing formal support services.

### Strength and limitations of the study

The limitations to our study include recall bias as participants may have not adequately remembered details of their experiences of abuse, and with services provided by formal support agencies. There was also the risk of social desirability bias where survivors may provide information in line with what is known to be socially acceptable. These limitations notwithstanding, the strength of our study rests in its contribution to evidence on the experiences of women survivors of domestic violence with formal support services in Ghana. Even though the study focused on urban Ghana, the findings may be useful for understanding issues of domestic violence against women in similar settings within, and out of Ghana where formal support and protective services for survivors of domestic violence are poorly delivered.

Several policy implications and recommendations emerge from this study. The study findings demonstrate that even though Ghana’s DV Act makes provision for justice and some social support interventions, only the police service and justice stipulations were operational. The main challenges that hindered the delivery of holistic services to DV survivors include inadequate training of court officials and police officers in the handling of gender-based violence, and the lack of infrastructure and operational logistics. Hence, there is the need for the government to increase its budgetary allocation to mandated institutions to facilitate in-service professional training programs that will equip personnel of the criminal justice system with skills to handle gender-based violence in accordance with international standards.

The root causes of DV – poverty and patriarchy should be given close attention through intensification of already running programmes on girl child education, women empowerment projects, mass public education and sensitization exercises to increase awareness – targeting the wider community with special attention on children at an early age. The adoption of a policy of giving integrated services at one stop centers will be effective in rendering protective services to survivors. These centers will ensure that victims of DV receive free medical care and accompanying medical reports, free legal assistance, and shelters. This will facilitate speedy recovery from their injuries and mitigate complications that might arise from delays in seeking medical treatment. Prompt medical attention would also ensure that good medical evidence is not lost to time, but adequately captured in reports to facilitate adjudication of DV cases.

## Conclusions

The experiences of DV survivors do not reflect the legal provisions of Ghana’s DV Act. Government under funding of formal services and negative gender norms are disempowering to survivors. NGOs are popular among women survivors of DV in Ghana for the education, legal, and material support they provide. A close collaboration between the government and NGOs could better mitigate DV in Ghana.

## Data Availability

The interviews and transcripts analyzed during the current study are not publicly available due to the risks in identifying participants, as complete anonymization could be difficult to guarantee. However, subsets of the data can be available from the corresponding author upon a reasonable request.

## Abbreviations

ADR: Alternative Dispute Resolution
DOVVSU: Domestic Violence and Victims Support Unit
CHRAJ: Commission on Human Rights and Administrative Justice.
DV: Domestic Violence
FIDA: International Federation of Human Lawyers
SES: Socio-economic Status

## References

[1] CEDAW Committee. CEDAW General Recommendation No. 19: Violence against women (Eleventh Session,1992). https://www.legal-tools.org/doc/f8d998/pdf/&ved=2ahUKEwi4r8KY2dX 1992. p. 1–6.

[2] Ortiz-Barreda G, Vives-Cases C (2013). Legislation on violence against women: overview of key components. Rev Panam Salud Publica.

[3] Byrnes A, Bath E (2008). Violence against women, the Obligation of due diligence, and the Optional Protocol to the convention on the elimination of all forms of discrimination against women—recent developments. Hum Rights Law Rev.

[4] Sarkin J (2018). A methodology to ensure that States adequately apply due diligence Standards and processes to significantly impact levels of violence against women around the World. Human Rights Q.

[5] Adomako Ampofo A (2008). Collective activism: the domestic violence Bill becoming Law in Ghana. Afr Asian Stud.

[6] Coker-Appiah D, Cusack K (1999). Breaking the silence & challenging the myths of violence against women and children in Ghana: report of a national study on violence.

[7] Ofei-Aboagye RO (1994). Altering the strands of the fabric: a preliminary look at domestic violence in Ghana. Signs: J Women Cult Soc.

[8] Bouilly E, Cross H, Rillon O (2016). African women’s struggles in a gender perspective (dossier). Rev Afr Polit Econ.

[9] Medie PA (2013). Fighting gender-based violence: the women’s movement and the enforcement of rape law in Liberia. Afr Affairs.

[10] Adams M, Tripp AM, Ferree MM (2006). Regional women’s activism: african women’s network and the African Union. Global feminism: transnational women’s activism, organizing, and human rights.

[11] Caracci G (2003). Violence against women. Int J Mental Health.

[12] Pickup F, Williams S, Sweetman C (2001). Ending violence against women: a challenge for Development and Humanitarian.

[13] Owusu Adjah ES, Agbemafle I (2016). Determinants of domestic violence against women in Ghana. BMC Public Health.

[14] Bhatta DN (2014). Shadow of domestic violence and extramarital sex cohesive with spousal communication among males in Nepal. Reproductive Health.

[15] Koenig MA, Stephenson R, Ahmed S, Jejeebhoy SJ, Campbell J (2006). Individual and contextual determinants of domestic violence in North India. Am J Public Health.

[16] Muluneh MD, Stulz V, Francis L, Agho K (2020). Gender based violence against women in Sub-Saharan Africa: a systematic review and Meta-analysis of cross-sectional studies. Int J Environ Res Public Health.

[17] Rubenstein BL, Lu LZN, MacFarlane M, Stark L (2020). Predictors of interpersonal violence in the Household in Humanitarian Settings: a systematic review. Trauma Violence & Abuse.

[18] Semahegn A, Mengistie B (2015). Domestic violence against women and associated factors in Ethiopia; systematic review. Reproductive Health.

[19] Krug EG, Mercy JA, Dahlberg LL, Zwi AB (2002). The world report on violence and health. The Lancet.

[20] Lasong J, Zhang Y, Muyayalo KP, Njiri OA, Gebremedhin SA, Abaidoo CS (2020). Domestic violence among married women of reproductive age in Zimbabwe: a cross sectional study. BMC Public Health.

[21] Adu-Gyamfi E (2014). Challenges undermining domestic violence victims’ access to justice in Mampong municipality of Ghana. JL Pol’y & Globalization.

[22] Rohn E, Tenkorang EY. Structural and institutional barriers to help-seeking among female victims of intimate Partner violence in Ghana. J Family Violence. 2022. 10.1177/10778012221137924 PMC11370201 36408705

[23] Lelaurain S, Graziani P, Monaco GL (2017). Intimate Partner Violence and help-seeking. Eur Psychol.

[24] Hage SM (2006). Profiles of women Survivors: the development of Agency in Abusive Relationships. J Couns Dev.

[25] Barnette OW (2000). Why battered women do not leave, Part 1:external inhibiting factors within society. Trauma Violence & Abuse.

[26] Barnett OW, Trauma (2001). Violence & Abuse.

[27] Aujla W (2021). It was like Sugar-Coated words: Revictimization when south asian immigrant women disclose domestic violence. Affilia.

[28] Rohn E, Tenkorang EY (2023). Structural and institutional barriers to help-seeking among female victims of intimate Partner violence in Ghana. J Family Violence.

[29] Amoakohene MI (2004). Violence against women in Ghana: a look at women’s perceptions and review of policy and social responses. Soc Sci Med.

[30] Ghana Statitiscal Service (2012). 2010 Population and housing census: Summary report of final results.

[31] Institute of Development Studies (IDS)., Ghana Statistical Services (GSS), Associates. Domestic Violence in Ghana: Incidence, Attitudes, Determinants and Consequences. Brighton2016.

[32] Darkwah AK, Prah M. Beyond domestic violence laws: women’s experiences and perceptions of protection services in Ghana (Regional evidence papers). CEGENSA; 2016.

[33] Aboagye EM, Effah KO, Asamoah RA. Fighting against domestic violence: the cases of Ghana and China. IUP Law Review. 2022;12(1).

[34] Issahaku PA (2016). Policy suggestions for combating domestic violence in West Africa. Int J Sociol Soc Policy.

[35] Minisry of Gender. Committee on the Elimination of Discrimination Against Women (2016). Response to list of issues and questions in relation to the combined Sixth and Seventh Periodic Reports of Ghana.

[36] Bonnes S (2016). Education and income imbalances among married couples in Malawi as predictors for likelihood of physical and emotional intimate partner violence. Violence Vict.

[37] Ogum Alangea D, Addo-Lartey AA, Sikweyiya Y, Chirwa ED, Coker-Appiah D, Jewkes R (2018). Prevalence and risk factors of intimate partner violence among women in four districts of the central region of Ghana: baseline findings from a cluster randomised controlled trial. PLoS ONE.

[38] Tenkorang EY, Owusu AY, Yeboah EH, Bannerman R (2013). Factors influencing domestic and marital violence against women in Ghana. J Family Violence.

[39] Forsythe N, Korzeniewicz RP, Durrant V (2000). Gender inequalities and economic growth: a longitudinal evaluation. Econ Dev Cult Change.

[40] Kim J, Pronyk P, Barnett T, Watts C (2008). Exploring the role of economic empowerment in HIV prevention. AIDS.

[41] Morrison A, Ellsberg M, Bott S (2007). Addressing gender-based violence: a critical review of interventions. World Bank Res Obs.

[42] Seedat M, Van Niekerk A, Jewkes R, Suffla S, Ratele K (2009). Violence and injuries in South Africa: prioritising an agenda for prevention. The Lancet.

[43] Fineman MA (2010). The vulnerable subject and the responsive state. EmoRy lJ.

[44] Kohn NA (2014). Vulnerability theory and the role of government. Yale JL & Feminism.

[45] Akotia CS, Anum A, Gender, Safdar S, Kosakowska-Berezecka N (2015). Culture, and Inequality in Ghana: an examination of Sociocultural determinants of gender disparity. Psychology of gender through the Lens of Culture: theories and applications.

[46] Buor D (2004). Gender and the utilisation of health services in the Ashanti Region, Ghana. Health Policy.

[47] Shabaya * J, Konadu-Agyemang K. Unequal access, unequal participation: some spatial and socio‐economic dimensions of the gender gap in education in Africa with special reference to Ghana, Zimbabwe and Kenya. Compare: A Journal of Comparative and International Education. 2004;34(4):395–424.

[48] Mitchell L (2011). Service users’ perceptions of the domestic violence and victims’ support unit, Ghana Police Service.

[49] Darkwah AK, Prah M. Beyond domestic violence laws: women’s expereinces and perceptions of protection services in Ghana (Regional evidence papers) 2016 [Available from: https://cegensa.ug.edu.gh/sites/cegensa.ug.edu.gh/files/Beyond%20Domestic.pdf.

[50] Cannon LM, Sheridan-Fulton EC, Dankyi R, Seidu A-A, Compton SD, Odoi A (2020). Understanding the healthcare provider response to sexual violence in Ghana: a situational analysis. PLoS ONE.

[51] Jamieson L, Sambu W, Mathews S. Out of harm’s way? Tracking child abuse cases through the child protection system at five selected sites in South Africa Cape Town. Children’s Institute, University of Cape Town; 2017.

[52] Meyer S (2016). Examining women’s agency in managing intimate partner violence and the related risk of homelessness: the role of harm minimisation. Glob Public Health.

[53] Stylianou AM, Pich C (2021). Beyond domestic violence shelter: factors Associated with Housing Placements for Survivors Exiting Emergency Shelters. J Interpers Violence.

[54] Greeson MR, Campbell R (2011). Rape Survivors’ Agency within the Legal and Medical Systems. Psychol Women Q.

[55] Cantalupo N, Martin LV, Pak K, Shin S (2006). Domestic violence in Ghana: the open secret. Geo J Gender & L.

[56] Yahaya MJ. Victims of domestic violence pay for medical services though it’s free. Daily Graphic. 2018 5th May, 2018.

[57] Carlson MJ, Harris SD, Holden GW (1999). Protective orders and domestic violence: risk factors for Re-Abuse. J Family Violence.

[58] Meinck F, Cluver LD, Orkin FM, Kuo C, Sharma AD, Hensels IS (2017). Pathways from family disadvantage via abusive parenting and caregiver Mental Health to Adolescent Health Risks in South Africa. J Adolesc Health.

[59] Sedziafa AP, Tenkorang EY, Owusu AY, Sano Y (2017). Women’s experiences of intimate Partner Economic abuse in the Eastern Region of Ghana. J Fam Issues.

[60] Grauwiler P, Mills LG (2004). Moving beyond the criminal justice paradigm: a radical restorative justice approach to intimate abuse. J Soc & Soc Welfare.

[61] McPhail BA, Busch NB, Kulkarni S, Rice G (2007). An integrative Feminist Model: the evolving Feminist Perspective on intimate Partner violence. Violence Against Women.

[62] Bassadien SR, Hochfeld T (2005). Across the public/private boundary: contextualising domestic violence in South Africa. Agenda.

[63] Dowuona-Hammond C, Atuguba RA, Tuokuu FXD (2020). Women’s survival in Ghana: what has Law got to Do with it?. SAGE Open.

[64] Ellsberg M, Arango DJ, Morton M, Gennari F, Kiplesund S, Contreras M (2015). Prevention of violence against women and girls: what does the evidence say?. The Lancet.

[65] Bennett L, Riger S, Schewe P, Howard A, Wasco S (2004). Effectiveness of Hotline, Advocacy, Counseling, and Shelter Services for victims of domestic violence:a statewide evaluation. J Interpers Violence.

[66] McLeod AL, Hays DG, Chang CY (2010). Female intimate Partner Violence Survivors’ experiences with Accessing Resources. J Couns Dev.

[67] Sullivan CM, Rumptz MH. Adjustment and needs of african-american women who utilized a domestic violence shelter. Violence Vict. (3):275–86. 7647048

[68] Tomm K (2019). Externalizing the problem and internalizing personal agency. J Systemic Ther.

